# Cross-talks between the kidneys and the central nervous system in multiple sclerosis

**DOI:** 10.22088/cjim.9.3.206

**Published:** 2018

**Authors:** Sahar Rostami, Mohammad-Sajad Emami-Aleagha, Maryam Ghasemi-Kasman, Abdolamir Allameh

**Affiliations:** 1 Department of Clinical Biochemistry, Faculty of Medicine, Tarbiat Modarres University, Tehran, Iran; 2 Cellular and Molecular Biology Research Center, Health Research Institute, Babol University of Medical Sciences, Babol, Iran

**Keywords:** Multiple sclerosis, Kidney, Central nervous system, Hormones

## Abstract

Multiple sclerosis (MS) is an inflammatory demyelinating disease, which is considered as a common autoimmune disorder in young adults. A growing number of evidences indicated that the impairment in non-neural tissues plays a significant role in pathology of MS disease. There are bidirectional relationship, metabolic activities and functional similarity between central nervous system (CNS) and kidneys which suggest that kidney tissue may exert remarkable effects on some aspects of MS disorder and CNS impairment in these patients compels the kidney to respond to central inflammation. Recently, it has been well documented that hormonal secretion possesses the important role on CNS abnormalities. In this regard, due to the functional similarity and significant hormonal and non-hormonal relationship between CNS and kidneys, we hypothesized that kidneys exert significant effect on initiation, progression or amelioration of MS disease which might be regarded as potential therapeutic approach in the treatment of MS patients in the future.

Multiple sclerosis (MS) is a chronic autoimmune disease of the central nervous system (CNS) which mainly affects young people worldwide (1). Numerous studies suggested that MS patients show several impairments including vascular, musculoskeletal, gastrointestinal, ocular (visual), mental, renal problems which lead to negative impacts on quality of life (2). In fact, the systemic inflammation and overproduction cytokines contribute to the pathogenesis of multiple organs dysfunction and failure (3). In addition, it has been shown that injury in one organ can lead to injury in a distant organ (4). In this line peripheral inflammatory factors such as TNF-α plays an important role in brain damage of animals with experimental autoimmune encephalomyelitis (EAE), as a model of MS. Indeed, dendritic spines and axonal buttons loss were observed 7 days after EAE induction. Moreover, synaptic instability also occurred before T-cell infiltration and microglia activation in the CNS. It seems that peripheral production of TNF-α leads to sensory and cognitive defects. Therefore, anything that increases peripheral but not central TNF-α production can potentially induce CNS damage (5). Recent studies have indicated that non-neural impairments might play an important role in MS initiation and progression. For instance Nouri et al., (2014) reported that overexpression of zonulin, as tight junction modulator protein, can increase intestinal permeability in EAE model. It has been suggested that entrance of intraluminal antigens into systemic circulation triggers antigen interaction with immune cells. Furthermore, increased intestinal permeability may lead to inflammation and morphological changes in ileum and jejunum cells and initiate multi-organ failure which causes the autoimmune responses (1). 

Several studies also describe the bidirectional relationship between the brain and kidneys. In this regard, after neurological dysfunction or renal failure, the pathological mechanism including alteration in microscopic anatomy, vasoregulation and hormonal pathways can cause chronic degeneration changes in both organs (3). The aforementioned examples are sufficed to hypothesize the possible functional relationship among kidneys, CNS and immune system in the initiation and progression of MS disorder. Undoubtedly, the kidneys exert important physiological functions in the human body such as removing waste products, electrolyte balance and blood pressure regulation. 

Besides, kidneys are involved in the production and secretion of several hormones including erythropoietin (EPO), renin, 1, 25-dihydroxyvitamin D and also a newly found anti-aging protein which is called Klotho (6). Based on the critical role of kidneys in homeostasis maintenance, one might speculate that, functional impairment in kidneys may lead to significant effects in the CNS. On the other hand, this hypothesis may provide the new therapeutic approaches for treatment of MS disease. In this regard, if the hypothesis is proven to be correct, monitoring of kidney function is necessary for MS patients and helps them to avoid progression of MS disease. It can also be helpful to use recombinant EPO and Klotho as anti-inflammatory and anti-aging hormones in order to compensate the impaired hormonal functions of kidneys. 

## Evaluation of the hypothesis

According to aforementioned data and based on the role of kidneys as endocrine/metabolic organ in expression of the secretory proteins, kidneys show a noticeable similarity to the choroid plexus of CNS. Interestingly, kidneys and choroid plexus share nearly 211 common proteins. Since the physiological role of choroid plexus in maintaining the chemical balance of cerebrospinal fluid (CSF) is analogous to the function of kidneys in blood, choroid plexus has been introduced as the ‘kidney’ of the brain (7). There are several studies proving the correlation between the brain and kidneys. For instance, patients with chronic kidney disease (CKD) are potentially susceptible to impairment of cognition. Furthermore, brain imaging studies demonstrated that white matter lesions occur in CKD patients. The possible explanation for brain lesions in CKD patients could be attributed to uremia, calcium-phosphate imbalance and inflammatory responses that have occurred over time after kidney damage (8). Acute kidney injury (AKI) is the other renal disorder leading to amplification of damage in brain through cytokine production and oxidative stress (9, 10). Secretion of pro-inflammatory chemokines (CXCL 1, 2 and 3) and cytokines (IL-1, 6, 10, 18, INF-γ and TGF-β) from kidney and activation of Toll like receptor 2 and 4 in astrocyte, microglia and neurons during AKI can contribute to the BBB disruption and brain local inflammation. This is in contrast to the traditional concept that the CNS is isolated from the immune system (9). Several studies also indicated that brain injury leads to changes in the kidney function. Cell death in the brain alters distant organs through hemodynamic instability, increase immunological complications and hormonal disturbance (4, 11, 12). It has been suggested that brain injury directly affects the kidneys by 3 mechanisms:

1) Enhancement of neural and renal sympathetic activity 2) Hypothalamo-pituitary axis 3) Intra cranial inflammatory cytokines cross the BBB through BBB-deficient areas into the systemic circulation and elevate the systemic inflammation and induce the oxidative stress process (13). Another study on progressive type of MS showed that the estimated glomerular filtration rate (eGFR) of patients is significantly lower than healthy individuals. Although reduction in eGFR revealed disruption in kidney function, but unexpectedly, their serum creatinine was normal or lower than normal. In the progressive type of MS disease, high volume of muscle mass is lost which leads to serum creatinine level decline. Therefore, this effect can mask the real changes in eGFR (14).

Additionally, there are significant hormonal-mediated cross-talks between CNS and kidneys in normal or abnormal status. In this context, the following provided evidences in support to hormonal relationship between kidneys and brain. 

## Renin angiotensin system (RAS)

RAS is an important hormonal system in cerebrorenal connection. Low blood pressure stimulates juxtaglomerular cells to secrete Renin. In addition, renin converts angiotensinogen to angiotensin I (Ang I). In turn, Ang I is cleaved to Ang II by angiotensin-converting enzyme (ACE). Although it is worth mentioning that variety of receptors has been found for Ang II on the surface of kidney, heart, brain and immune cells. Ang II receptors initiate inflammation and increase blood pressure.

 Furthermore, ACE and Ang II receptor type 1 (AT1R) increase antigen presenting cell and making central inflammatory response(15). Salt intake and sodium ion accumulation can stimulate renal and cerebral RAS, which connect to each other by afferent and efferent sympathetic nerves. The reaction has been termed the “reno-cerebral RAS axis“. This system activates NADPH oxidase and increases free radical production, inflammatory cell population and blood brain barrier (BBB) permeability (16, 17) In patients with CKD disease, kidney mass, enzymes activity and secreted factor significantly decreased. Accordingly, the amount of enzymes such as 1-alpha-hydroxylase (which activates vitamin D) and hematopoietic hormone EPO are influenced. Thereby reducing vitamin D and EPO levels, the renin expression and RAS activity increase and stimulate immune system and initiate neuronal lesion (18).

## Erythropoietin (EPO)

Although brain, liver and uterus are able to synthesize EPO, kidney is the main source for secretion of EPO which acts systemically. Unlike the kidneys, there is paracrine EPO/ EPO receptor in brain that acts independently from the endocrine system (19). In the CNS, EPO acts as anti-apoptotic, anti-inflammatory, anti-oxidant and neurotropic factor and exerts important role in oligodendrocyte progenitor cells proliferation (20, 21). 

Therefore, EPO deficiency or insufficiency has adverse effects on human brain. Former assumption implies that large molecule such as EPO cannot transfer across the BBB. Brines et al. (2000) indicated that high dose intravenous (IV) injection of recombinant EPO can cause EPO passing through BBB. 

These results suggesting EPO as neuroprotective agent, is able to alleviate the severity of disease in animal models of focal brain ischemia, concussive brain injury, EAE and kainate-induced seizures. In fact, EPO delays the TNFα level overexpression, ameliorates gliosis and reduced the infiltration of inflammatory factors into spinal cord (20, 22). De-he et al. indicated that serum EPO level in MS patients complicated with anemia was lower than iron deficiency anemia (IDA) (23). Of note, because of kidney failure, EPO secretion decreased in these patients. According to the earlier data, it is plausible that EPO consumption is a compensatory response to immune stimulation or inflammation in MS patients. 

## Vitamin D

Calcitriol or 1, 25-dihydroxy vitamin D is the active form of vitamin D that is produced in proximal tubules of the kidney by 1-alpha hydroxylase and supports the endocrine actions of vitamin D. Calcitriol is secreted into the systemic circulation and exert its effects in various tissues (24). It is beyond doubt that calcitriol has direct effect on brain and immune system. Immune cells such as macrophages, monocytes and dendritic cells express vitamin D receptor (VDR) on their surface as well as all types of the CNS cells including neurons, oligodendrocytes and astrocytes. In addition, calcitriol binds to VDR and decreases the secretion of cytokines and regulates adaptive immunity in multiple ways (25). Studies on MS patients have shown that the expression level of vitamin D activator enzyme such as 1-alpha hydroxylase is lower than normal persons. Based on the role of vitamin D in immune system modulation, these persons may be susceptible to autoimmune diseases (26). Furthermore, it has been shown that renal inflammation is associated with reduction in serum vitamin D metabolites. Besides, previous studies using vitamin D analogs have indicated that the endocrine vitamin D system may also contribute to immunomodulatory responses. Increase in serum 1, 25-dihydroxy vitamin D or 25-hydroxy vitamin D resulted in lower renal inflammation (24, 27). As mentioned previously, people with kidney dysfunction have lower expression of 1-alpha hydroxylase, so it seems that the risk of MS disease is higher than normal persons in these patients. 

## Klotho (KL)

KL is an anti-aging protein which is mainly synthesized in kidneys, brain, parathyroid gland and heart. Among these tissues, the choroid plexus and kidneys are the most important regions for KL secretion. Basically two forms of KL were detected in the human body, including transmembrane KL (T- KL) and secreted Klotho (S-KL). The T- KL is a co-receptor for fibroblast growth factor 23 (FGF23) signaling. In this way, FGF23 binds to the FGF23 receptor with much higher affinity than FGF23 receptor or Klotho alone. 

This interaction suppresses 1-alpha-hydroxylase expression and 1, 25-dihydroxyvitamin-D production. The extracellular domain of T-KL is proteolyzed by ADAM10 and ADAM17 which causes conversion of T-KL to the other form of KL. These fragments that are released to circulation system have been termed S-KL. S-KL have anti-inflammatory and antioxidant activities and play an important anti-inflammatory role in the brain and kidney (28, 29). 

 Emami Aleagha et al. in 2015 demonstrated that the CSF level of KL in patients suffering from relapsing-remitting MS (RRMS) is significantly lower than control group (30). In 2016, they reported an evaluation of KL concentration in the serum of RRMS with prolonged duration is higher than control and new cases of MS disease. They suggested that, when MS disease lasts for a longer period, the regenerative pathways need to be more activated and so it might be responsible for increasing the serum level of KL (31). It means that the enhancement in S-KL production by the kidneys is a compensatory response to the neurodegenerative condition. Furthermore, the cleavage of T-KL for increasing the S-KL concentration, not only helps the immune system regulation, but also prevents the decline of 1, 25-dihydroxyvitamin-D. 

## How to test the hypothesis?

Several strategies are possible to test this hypothesis. The first one is the assessment of renal function by performing routine laboratory test on the serum and urine. In this approach, kidney response to the CNS inflammation in MS patients can be assessed with the evaluation of the level of renal endocrine hormones such as KL and EPO. Since renal biopsy operation from MS patients is not ethical, so induction of EAE as a conventional model of MS disease is considered as the best strategy to study the inflammatory condition.

 Structural alteration and the expression levels of aforementioned genes in the CNS and kidney in response to central inflammation can be evaluated with EAE model. CKD induction in rodents before EAE immunization is another way to determine susceptibility to EAE induction and evaluate intensity of lesions.

## Consequences of the hypothesis

On the basis of similarities in hormonal activities in kidneys and CNS as well as the association between kidneys and brain in hemodynamic, hormonal and sympathetic aspects, we predict that pathophysiological alterations in the CNS can be led to dramatic response in the kidney tissue of MS patients. 

These responses may reduce the severity of the CNS injuries or increase the lesion area. In this regard, it is plausible that impairment in kidney's secretory functions might contribute to brain dysfunction in MS patients and vice versa. In contrast, it is also possible that the aforementioned renal responses by the kidneys in MS patients can exert the beneficial effects on the improvement of the disease. The metabolic cross-talk between kidneys and CNS is not limited to endocrine hormones but corroborated with factors such as KL and vitamin D.

## Conflict of Interest:

The authors declare no conflict of interest.
